# The Therapeutic Effect of Myrrh (*Commiphora molmol*) and Doxorubicin on Diethylnitrosamine Induced Hepatocarcinogenesis in Male Albino Rats

**DOI:** 10.31557/APJCP.2021.22.7.2153

**Published:** 2021-07

**Authors:** Hend Mohamed Anwar, Asmaa M. Moghazy, Amany Abd Elhameid Elhameid Osman, Amina A. S. Abdel Rahman

**Affiliations:** 1 *Department of Biochemistry, National Organization of Drug Control and Research (NODCAR), Giza, Egypt. *; 2 *Department of Hormonal Evaluation, National Organization of Drug Control and Research (NODCAR), Giza, Egypt. *; 3 *Department of Zoology, Faculty of Women for Arts, Science and Education, Ain shams university, Asmaa Fahmy Street, Heliopolis, Cairo, Egypt. *

**Keywords:** Diethylnitrosamine-, Hepatocellular carcinoma, Myrrh (C. molmol), Doxorubicin

## Abstract

**Background:**

This study was conducted to assess the therapeutic effect of Myrrh on Diethylnitrosamine (DEN)-induced hepatocarcinogenesis (HCC) in male albino rats.

**Methods:**

Fifty male albino rats were divided into five groups (10 rats each). Group 1 (control group) received distilled water. Group 2 (positive control) was injected intraperitoneally with DEN (55 mg/kg b.w) twice a week for two weeks, while group 3 (DOX) received doxorubicin i.p (10 mg/ kg b.w) after concomitant with DEN twice a week for four weeks. Groups 4 and 5 received a low dose of Myrrh (250 mg/kg b.w) and a high dose of Myrrh (500 mg/kg b.w) respectively daily for four weeks after the induction with DEN. The sera were used to estimate the liver enzymes (ALT, AST, and ALP), Alpha-fetoprotein (AFP), Total antioxidant capacity (TAC), and Tumor necrosis factor-ἁ (TNF-ἁ). Also, the liver tissues were collected to determine the oxidative stress markers in addition to the histopathological and immunohistochemical investigations.

**Results:**

The results showed that the induction of DEN causes a significant increase in the level of liver enzymes (ALT, AST, and ALP), AFP and TNF-ἁ as well as produce oxidative stress indicated by increasing of malondialdehyde (MDA) with the reduction in TAC and glutathione (GSH). Meanwhile, there are noticeable histopathological lesions with loss of hepatic architecture. This was accompanied by a significant increase of immunohistochemical markers; Caspase-3, vascular endothelial growth factor (VEGF), transforming growth factor β1(TGF- β1), and carcinoembryonic antigen (CEA) percentage area. The treatment of DEN rats with DOX reduced the alterations in most parameters. A marked amelioration of all parameters in a dose-dependent manner of Myrrh to the values almost near to those of the control group.

**Conclusion:**

Our data revealed that Water extract of Myrrh (*C. molmol*) has a potential therapeutic effect in attenuation of HCC induced DEN.

## Introduction

Hepatocellular carcinoma (HCC) is the sixth most common tumor and the fourth leading cause of cancer-associated death worldwide (Bray et al., 2018) and accounts for 80 – 90 % of all main liver cancers (Vogel et al., 2018). Many factors cause cirrhosis that increases the risk of HCC (Fong et al., 2010) as viral infection with the hepatitis B or hepatitis C (Arzumanyan et al., 2013), alcoholism (Scoccianti et al., 2016), congenital metabolic diseases, diabetes, and smoking (Chuang et al., 2009) along with exposure to carcinogenic compounds as aflatoxins (Cai et al., 2020). This is a set of mycotoxins created by the Aspergillus flavus in cereals like corn and peanuts due to storage in warm and moist and is a common cause of carcinogenesis (Dai et al., 2017). DEN, a genotoxic compound, has a crucial role in generating reactive oxygen species (ROS) that induce oxidative stress in addition to impairment in the nuclear enzymes related to (DNA) repair/replication by the formation of alkyl DNA adducts, then causing chromosomal aberrations and finally promote the development of HCC (Dar et al. 2019). Therefore, DEN is used for inducing liver cancer in rodents as an experimental model of human HCC (Elguindy et al., 2018). Meanwhile, DOX is one of the primary anthracyclines, which isolated from Streptomyces (Frengova et al., 2006). It has been considered as one of the antitumor therapeutic drugs (Luk et al., 2007). Unfortunately, the disadvantage of using DOX is cardiomyopathy that becomes severe with dependent cumulative doses (Chatterjee et al, 2010). In HCC chemotherapy, the administration of DOX revealed limited potential benefits as the tumor shrinkage, drug resistance and fractional reactions occur in 30 – 70 % of patients when the drug was given by the hepatic artery route and accompanied with some form of a hepatic artery-blocking agent (Rohman et al., 2019). 

As a result, new strategies were conducted to search for an alternative anticancer agent with more efficacy and fewer adverse effects. Meanwhile, Natural compounds from plants are known as phytochemicals have anticancer properties. They act via controlling molecular pathways which are associated with cancer growth by improving antioxidant status, carcinogen deactivation, inhibiting proliferation, induction of apoptosis (Choudhari et al., 2020) as well as inhibition of mutagenesis and epigenesis of DNA (Badr et al., 2017). Though, Myrrh (*C. molmol*), a popular natural medicine, is an oleo gum-resin obtained from the stem of *C. molmol* (Nees) Engl. (Burseraceae) and composed of 2-8% essential volatile oil, 23- 40% alcohol soluble resin, 40-60% water-soluble gum, and a bitter principle of 10- 25% (Trease and Evans 1989). It has several pivotal therapeutic effects as anticancer activity (Shoemaker et al., 2005) as well as cytotoxic activity against MCF-7 and HS-1 cells (Chen et al., 2013), and antioxidant (Dolara et al., 2000). The anti-inflammatory effect (Steinman 2017), hypolipidemic agent (Moustafa et al., 2018), cardioprotective (Hu et al., 2016), and anti-bacterial activity (Khalil et al., 2020) were also reported. Furthermore, Myrrh has antipyretic, analgesic, and antiseptic activities (Shalaby and Hammouda 2014) antirheumatic (Su et al., 2015), antiparasitic (El-Sayad et al., 2017), hypotensive (Abdul-Ghani and Amin 1997), antimicrobial (Mahboubi and Mohammad, 2016), and a strong antifungal activity (Perveen et al., 2018). Many studies confirmed in vitro the cytotoxic activity of Myrrh on human tumor cell lines especially HCC and induce a high level of apoptosis (Ramadan et al., 2017; Khalil et al., 2020) while in vivo, it reduces the rate of tumor growth (Mahmoud et al., 2017). Consequently, this study was conducted to assess the hepatoprotective effect of *C. molmol* resin water extract against DEN-induced early-stage hepatocarcinogenesis in male albino rats via its modulator role on tumor markers as AFP, TNF-α, CEA, Angiogenic factors, apoptosis, oxidative stress as well as histopathological alterations.

## Materials and Methods

*Chemicals*

- N-Diethyl Nitrosamine (DEN) was purchased from Sigma Aldrich (St. Louis, MO, USA) (CAS no. 55-18-5).

- Doxorubicin was delivered from Pharmacia Italia S.P.A. Milan, Italy.

- Myrrh resin (*C. molmol*) was purchased from a local trader, prepared, crushed then 100 g of hygienic crushed myrrh resin was added to 1,000 ml of distilled water. The blend was heated to boiling at 100°C for 30 min. The mixture was centrifuged, filtered, frozen, and then lyophilized according to (Liu et al., 2015).

*Animals*

Fifty Adult healthy male Wistar albino rats (Rattus norvegicus), weighing 100-120 grams was used in this experiment. The rats were obtained from National Organization for Drug Control and Research (NODCAR), Giza, Egypt. They were housed randomly in plastic cages with mesh wire covers under standard conditions (Temperature 25± 2^o^C, 12 h light-dark cycle, and humidity 50%–70%). Animals were accessed standard rat diet of pellet and water ad libitum. Care and use of the animals were conducted under the supervision of the Animal Ethics Committee of The National Organization for Drug Control and Research (NODCAR)(REC-Protocol Number: NODCAR II 57 19).

*Experimental design*

After two weeks of acclimatization, rats were randomly divided into five groups of ten rats each, as follows:

- Group (1) (Normal control); received distilled water 0.5 ml/kg. b.w. p.o. daily.

- Group (2) (positive control); received DEN only at a dose of (55mg/kg b.w. twice a week for 2 weeks according to (Li et al., 2017).

- Group (3) (DEN+DOX); received DOX (10 mg/kg. b.w intraperitoneally) twice a week for 4 weeks after injection of DEN with the previous dose according to (Pieniążek et al., 2013)

- Group (4) (DEN+ Myrrh 250); treated with a low dose of Myrrh (250 mg/kg b.w. p.o daily) for 4 weeks after the injection of DEN with the previous dose.

- Group (5) (DEN+ Myrrh 500); treated with a high dose of Myrrh (500 mg/kg b.w. p.o daily) for 4 weeks after the induction of DEN with the previous dose. 

The experiment was maintained for 6 weeks. At the end of the experimental period, all animals were fasted overnight, sacrificed by deep isoflurane inhalation. Blood samples were collected from the retro-orbital plexus vein. Serum was separated by centrifugation at 3,000 rpm for 15 min. at 4^o^C and stored at -20°C until further biochemical analysis. 

*Biochemical analysis*

*Measurement of serum biomarkers*

The activity of aspartate aminotransferase (AST), alanine aminotransferase (ALT), and alkaline phosphatase (ALP) in serum of rats was determined using reagent kits purchased from Bio diagnostics. (Cat no. AL 1031, AS 1061). Meanwhile, ALP was assayed using biochemical kits supplied by Spin react, Barcelona, Spain.

Serum levels of AFP, TAC, and TNF-ἁ were assayed by the ELISA kit according to the manufacturer`s instruction (Abia Ref. DK.045.01.3 and Sino Gene Clon Ref. SG20127 respectively).

*Oxidative stress and antioxidant activity*

Liver tissues were isolated, washed in ice-cold isotonic saline, then let dry between two filter papers, crushed into small pieces, homogenized in ice-cold 1.15% KCl to make 10% (w/v) homogenate with Glass-Col motor-driven homogenizer (USA) for the determination of MDA as a marker of lipid peroxidation. The other portion of the liver tissue was homogenized in ice-cold 5% sulfosalicylic acid to make 10% (w/v) homogenate for the estimation of GSH.

*Histological and immunohistochemical investigation*

Samples of liver were washed in phosphate buffer saline (PBS) immediately after sacrifice and fixed in 10% formalin, then dehydrated in ascending grades of alcohol, cleared in xylene, and embedded in paraffin wax (congealing point 58–60°C). Tissue sections were cut at 5- 6 μm using a Rotatory Microtome and mounted on glass slides. After deparaffinization in xylene, tissue sections were stained with Hematoxylin and Eosin (H&E) according to the standard procedure (Bancroft and Gamble, 2007). For immunohistochemical investigation, 4 mm serial histological sections from formalin-fixed paraffin-embedded blocks of liver tissue were dewaxed in xylene, rehydrated through graded alcohols, immersed in 10 mMTris and 0.5 M EDTA at pH 9.0, and were finally microwaved twice for 5 min each. Subsequently, the sections were incubated with 3% H_2_O_2_ for 10 min to block endogenous peroxidase activity. The sections were then incubated overnight at 4 °C with anti-cleaved caspase-3 monoclonal antibody (MA5-11516, 1:400, NASDAQ: INVGN, Carlsbad, California, USA) anti-VEGF mouse monoclonal antibody mAb (GB14165; 1:200, Service biotechnology Co., Ltd, Olympia, USA), anti-TGF-β1 rabbit polyclonal Ab (GB11179; 1:500, Service biotechnology Co., Ltd, Olympia, USA) and anti-CEACAM5 polyclonal antibody (PA5-88533; 1:100, NASDAQ: INVGN, Carlsbad, California, USA). The bound antibody was visualized using a 3,3’-diaminobenzidine (DAB) substrate kit (Vector Laboratories, Burlingame, CA). Cells were counterstained with hematoxylin. The specificity of the technique was assessed by negative controls. Bound antibody was detected by the avidin-biotin-peroxidase complex method, using a commercial kit as recommended by the manufacturer (Vestastain ABC Elite kit; Vector, Burlingame, CA). 3,3’-diaminobenzidine tetrahydrochloride was used as the chromogen.

*Image Morphometry*

The morphometric analysis was performed at the Pathology Department, National Research Center using the Leica Qwin 500 Image Analyzer (LEICA Imaging Systems Ltd, Cambridge, England,) to measure the area (expressed in µ m^2^) and area percentage of nuclei and cytoplasm in each cell for calculation of nuclear-cytoplasmic ratio (N/C ratio) in each group. The results appear automatically on the monitor in the form of area, area fraction ad area percentage according to (Rahman and Itakura 1996)

*Image analysis*

A semi-quantitative estimation of Caspase 3, VEGF, TGF-β1, and CEA based on the staining intensity using application immunoreaction by Color extraction software (2020). The percentage of positive cells was done to evaluate the labeling index, where 5-8 fields per specimen were randomly selected. Positive cells were counted in sequential high-powered fields (X400) and the results were expressed as the mean number of positive cells per limited surface area. 

*Statistical analysis*

For biochemical parameters, data were expressed as means ± S.E (n =10 rats) values in the different groups. Statistical differences between groups were evaluated by one-way analysis of variance (ANOVA) followed by Dunnett using Graph pad prism version 5.1. P <0 .05 was considered significant.

For histopathological analysis, data were represented as mean ± S.E (n= 3) and analyzed using one-way analysis of variance by the SPSS for Windows software, version 16.0 to compare all groups. Once a significant F test was obtained, a post-hoc-least significant difference analysis was performed with the signifi¬cance level of p < 0.05.

## Results

*Biochemical analysis*

*Effect of water extract of C. molmol and DOX on diagnostic markers of liver function*

The administration of DEN to rats produced an acute hepatic injury manifested by a significant increase (p<0.05) in ALT, AST, and ALP with percentage changes (51.7%, 65%, and 38.9 % respectively) when compared with a control group. While treatment with DOX as a classical drug, significantly (P < 0.05) decreased the levels of liver enzymes when compared with the DEN group. Also, the supplementation of either 250 mg/kg or 500 mg/kg doses of water extract of *C. molmol* ameliorated the liver injury as evidenced by a reduction in liver enzymes in a dose-dependent manner when compared with the DEN group as shown in Table 1.

*Effect of water extract of C. molmol and DOX on the levels of AFP, TAC, and TNF-ἁ*

The level of AFP and TNF-ἁ in serum showed a significant increase in the rats treated with the DEN with percentage changes (711.3%, and 25.3% respectively), while the level of TAC was significantly decreased in the same rats with percentage change (-75.65%) when compared with the control group at (P< 0.05). On the other hand; treatment with DOX significantly decreased the levels of AFP and TNF-ἁ and increase TAC level when compared with the DEN group (P< 0.05). However, treatment with *C. molmol* at 250 and 500 mg/kg. b.w. doses improved the previous parameters when compared with the DEN group in a dose-dependent manner as shown in Table 2.

*Effect of water extract of C. molmol and DOX on the levels of liver oxidative stress MDA and GSH*

Hepatic MDA level, as a marker of lipid peroxidation was significantly increased in rats treated with DEN with percentage change (107.22%), While the level of GSH was significantly decreased with percentage change (-51.38%) when compared with the control group at (p< 0.05). On the other hand; treatment with DOX reduced the hepatic MDA and increased GSH when compared with the DEN group at (P< 0.05). Moreover, treatment with *C. molmol* at 250 and 500 mg/kg. b.w doses showed a significant (p<0.05) reduction in hepatic MDA and increased in GSH when compared with the DEN group in a dose-dependent manner. Table 3.

*The histopathological examination*

**Figure 1:**
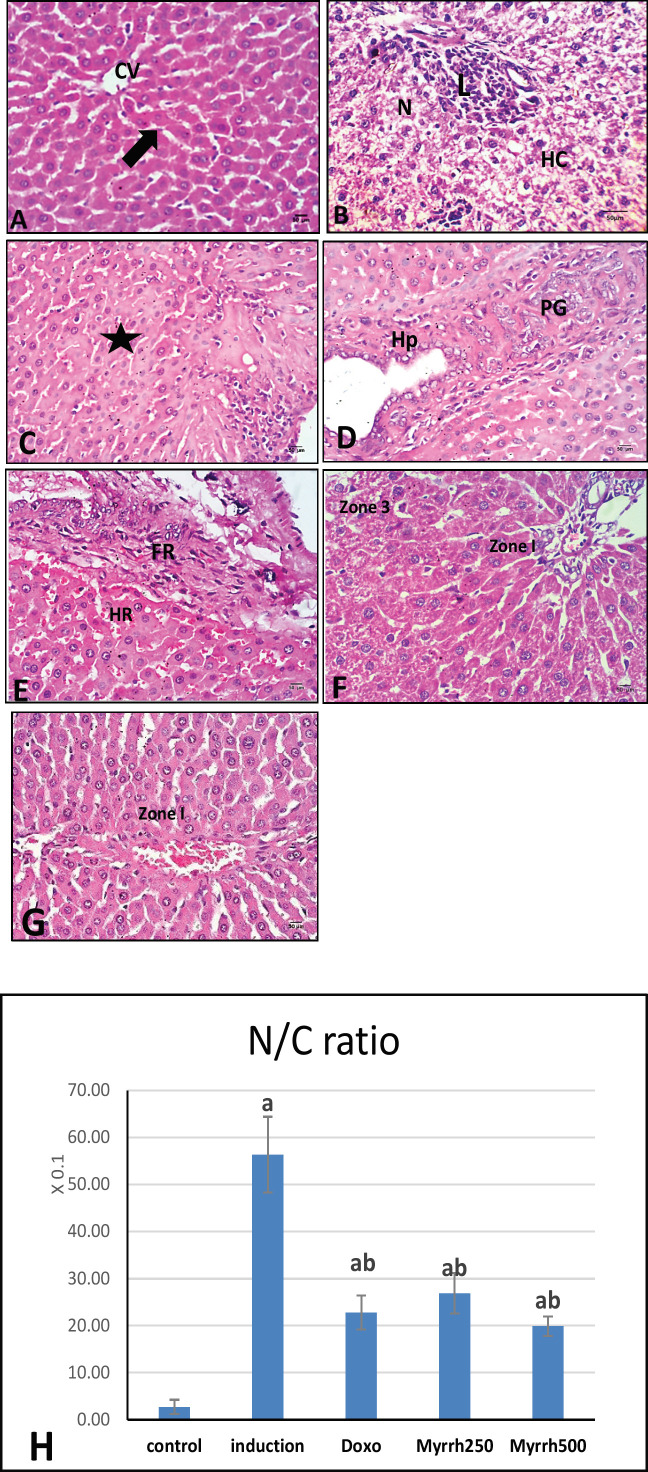
The photomicrographs of hepatocytes. (A) Control rats showing normal liver architecture. Central vein (CV), hepatocytes (arrow). (B) DEN group showed hydropic change (HC), necrotic cells (N), lymphocyte inflammation patches in portal area (L). (C) many mitotic cells and loss of cell boundaries and radiation arrangements (black star). (D) hyperplasia in bile duct (Hp) and pseudo-glandular pattern (PG). (E) DEN+DOX, liver showing massive fibrosis (FR) and hemorrhage in blood sinusoids (HR). (F, G) DEN+ Myrrh 250 and 500 mg/kg respectively, hepatocytes showed remarkable improvement in zone (1) with partial hydropic changes in zone 3. (H&E x400); (H): Chart illustrating the percentage of N/C ratio in hepatic tissue sections of all groups. The values are presented as mean ± standard error and significance (p< 0.05) compared to a con­trol group, (a) DEN (b) DEN+ DOX (c) DEN+Myrrh 250 mg/kg (d) DEN+Myrrh 500 mg/kg

The histological examinations mostly supported the result of serum enzymes, tumor, and inflammatory markers. Liver sections of the control group stained with H&E showed normal hepatic architecture. Hepatic lobules showed anastomosing cords of hepatocytes radiating from the central vein toward the periphery of the lobule and the portal triad (hepatic artery, portal vein, and bile duct (Figure 1 A). The nucleus is centrally located, and most hepatocytes are mononuclear while binuclear hepatocytes are often found. The blood sinusoids (b.s) are lined by fenestrated endothelium and Kupffer cells rest in it while stellate cells are found in space of Disse. In a contrast, the sections of the liver-induced DEN showed loss of radiating hepatocytes architecture and forming nests of pleomorphic atypical hepatocytes. It also evidenced the features of necrosis and vacuolar hydropic changes with intralobular lymphocytic infiltration (Figure 1 B). The nuclear size and hepatocytes also showed wide variation with increasing N/ C ratio as illustrated in (Figure 1H) and mitotic index with loss of cell boundaries and radiation arrangements with relative giant hepatocytes and eosinophilic cytoplasm as compared to the normal liver (Figure 1C). 

It also revealed the dilatation and proliferation of bile duct and intranuclear vacuoles, with pseudo-glandular arrangements and hyperplasia due to change of growth pattern (Figure 1D). While administration of rats with chemotherapy drug DOX was also examined for the comparison of the efficacy of Myrrh. It revealed relative histopathological changes accompanied by fibrotic areas (Figure 1E). The anti-cancerous property of Myrrh was reducing the severity of hepatic lesion in a dose-dependent manner. The treatment, with either two doses of Myrrh concomitant with DEN, was simply proportional to the dose resulted in a significant amelioration in histological profile, in zone 1 noticeable recovery in the liver architecture with restoring of radiating hepatocytes, central vein with slight sinusoidal and periportal infiltration (Figure 1F and G). The result also revealed statistically non-significant differences (P>0.05) in (N/C ratio) between Myrrh doses and DOX but highly significant with the DEN group.

*Immunohistochemical investigation:*

*Effect of water extract of C. molmol and DOX on the expression of caspase-3, VEGF, TGF-β1, and CEA*

**Figure 2:**
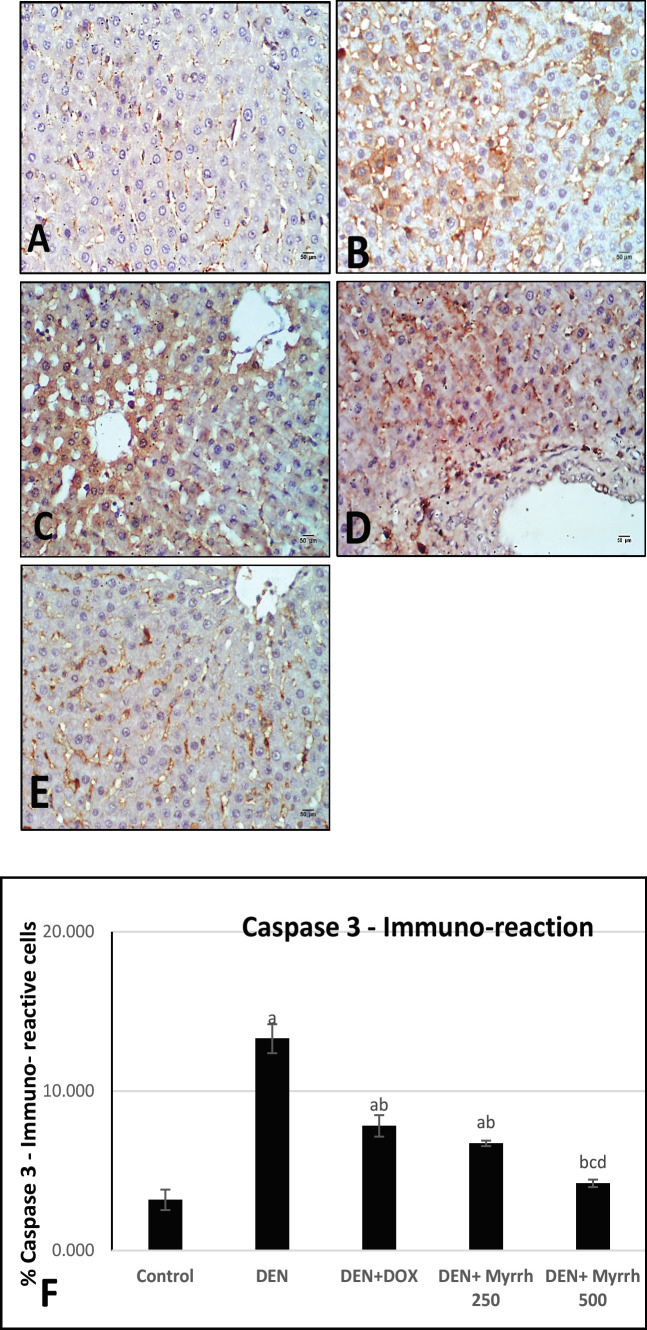
Photomicrographs of Hepatocytes Caspase-3 Immunohistochemistry. (A) Control rats show few Caspase-3 immunoreactive cells. (B): DEN group show a wide immunoreactive distribution. (C) DEN+DOX shows increase in Caspase-3 immunoreaction. (D-E): DEN+ Myrrh 250 mg/kg and 500 mg/kg, liver show relative decrease caspase-3 immunoreaction. (Caspase-3 X400). (F): Chart illustrate the percentage of Caspase-3 immunoreaction in hepatic tissue sections of all groups. The values are presented as mean ± standard error and significance (p < 0.05) compared to a con­trol group, (a) DEN (b) DEN + DOX (c) DEN+ Myrrh 250 mg/kg (d) DEN+ Myrrh 500 mg/kg

**Figure 3:**
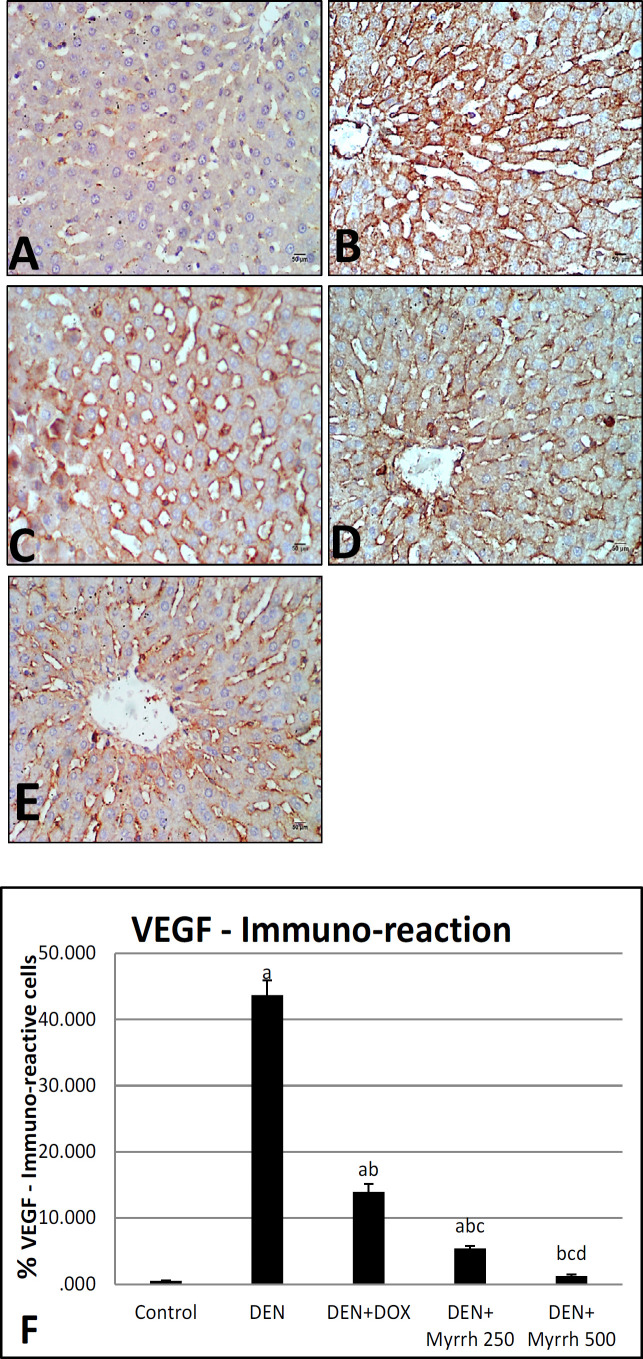
Photomicrographs of Hepatocytes VEGF Immunoreactivity. (A) Control rats show a few VEGF immune-reactive cells. (B) DEN group show intense staining distribution immunoreaction. (C) DEN+DOX shows a decrease in VEGF immunoreactivity. (D-E) DEN+ Myrrh 250 mg/kg and 500 mg/kg respectively, liver show a marked decrease in VEGF immunoreaction. (VEGF X400). (F) Charts illustrate the percentage of VEGF immunoreaction in hepatic tissue sections of all groups. The values are presented as mean ± S.E and significance (p < 0.05) compared to a con¬trol group, (a) DEN (b) DEN + DOX (c) DEN+ Myrrh 250 mg/kg (d) DEN+ Myrrh 500 mg/kg

**Figure 4:**
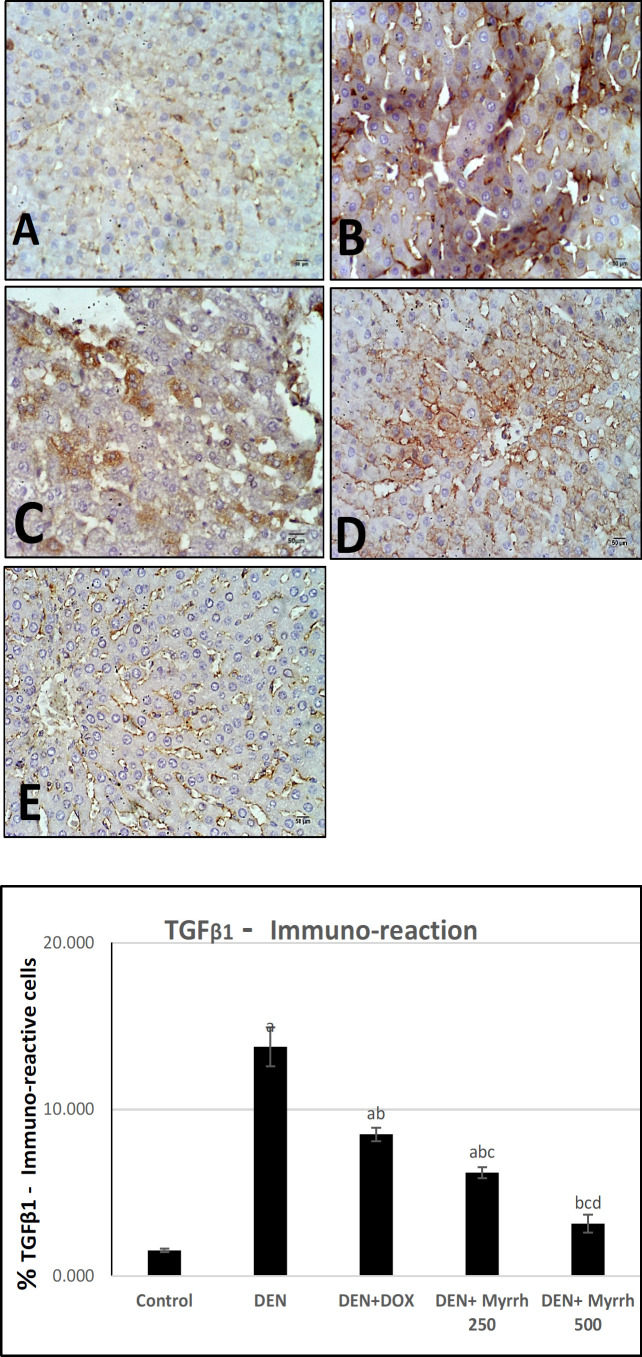
Photomicrographs of Hepatocytes TGF-β1 Immunoreaction. (A) Control rats show a few TGF-β1 immunoreaction. (B) DEN group showing wide distribution of TGF-β1 cells. (C) DEN+DOX. group, rats show a decrease in TGF-β1 immunoreaction (D-E) DEN+ Myrrh 250 mg/kg and 500 mg/kg respectively, rats liver show decrease TGF-β1 immunoreaction (TGF-β1 X400). (F): Chart illustrate the percent of TGF-β1 immunoreaction in hepatic tissue sections of all groups. The values are presented as mean ± S.E and significance (p < 0.05) compared to a con­trol group, (a) DEN (b) DEN + DOX (c) DEN+ Myrrh 250 mg/kg (d)DEN+ Myrrh 500 mg/kg

**Figure 5:**
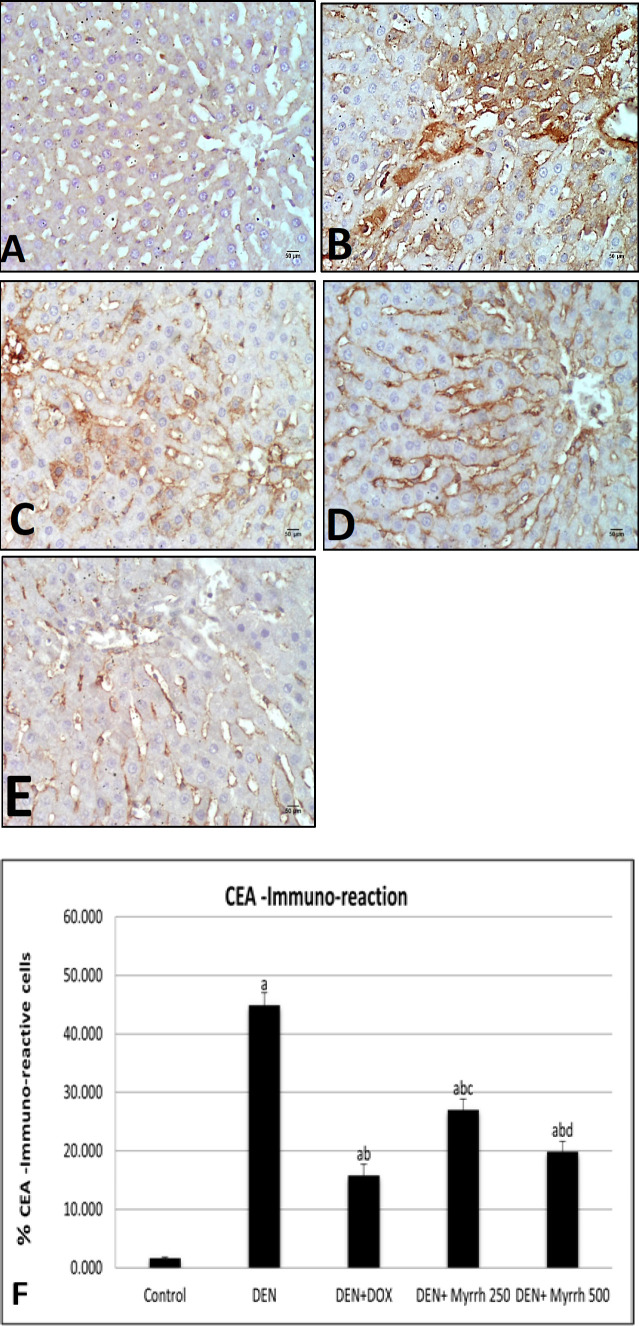
Photomicrographs of Hepatocytes CEA Immunohistochemistry. (A) Control rats show normal CEA immuno-reaction. (B) DEN group Shows a wide distribution of immuno-reactive cells. (C) DEN+DOX group, rats show a marked increase CEA immunoreactive cells. (D, E) DEN +Myrrh 250 mg/kg and 500 mg/kg respectively, liver show relatively increase CEA immunoreaction (CEA X400). (F): Chart illustrate the percentage of CEA immunoreaction in hepatic tissue sections of all groups. The values are presented as mean ± S.E and significance (p < 0.05) compared to a con¬trol group, (a) DEN (b) DEN +DOX (c) DEN+ Myrrh 250 mg/kg (d) DEN+ Myrrh 500 mg/kg

Active Caspase-3 positive cells, as a marker for apoptosis in the cytoplasm, were observed by immunohistochemistry in the liver sections of treated groups as compared to the positive group (DEN group) (Figure 2). Angiogenesis was visualized by immunostaining of liver sections with VEGF (Figure 3). The positive TGF- β1 staining was demonstrated in liver tissues (Figure 4). The effects of Myrrh extract on CEA immunoreactivity cells of DEN-induced rats are illustrated in (Figure 5). Immunoreactivity of Caspase-3, VEGF, TGF- β1, and CEA in the DEN group exhibited a significant (P< 0.05) increase when compared with the control group (Figures 2, 3, 4 & 5 H) respectively. The supplementation of either 250 mg/kg or 500 mg/kg dose of Myrrh extracts significantly (P< 0.05) decreased Caspase-3, VEGF, TGF- β1, and CEA immunoreactivity cells as shown in (Figures 2, 3, 4, 5 C, D and E) respectively while there is no significance between two doses of Myrrh and DOX group.

**Table 1:** Effect of Water Extract of *C. molmol *and Doxorubicin on the Levels of ALT, AST, and ALP

	Parameters					
Groups	ALT (U/L)		AST (U/L)		ALP (U/L)	
	Mean ± SE	% change	Mean ± SE	% change	Mean ± SE	% change
Control	19.33±1.2	…….	48.93±0.5	………	102±6.4	……
DEN	29.33±2.6*	51.70%	80.73±0.8*	65%	141.7±2.4*	38.90%
DEN+DOX	14.67±1.4^#^	-49.98%	59.7±1.8^#^	-26%	120.7±1.2^#^	-14.80%
DEN+ Myrrh 250	23.33±0.88^#^	-20.50%	74±2.0^#^	-8.30%	125.7±1.7^#^	-11.29%
DEN+ Myrrh 500	16 ±2.1^#^	-44.70%	47±1.5^#^	-41.80%	101±5.6^#^	-28.70%

Data are expressed as Mean ± SE (n = 10 rats), Means with different superscript letters are significantly different at p < 0.05. Data are analyzed with one-way ANOVA followed by the Dunnett test at p < 0.05. *significant difference from control and ^#^ significant difference from induction with DEN group at p < 0.05.

**Table 2.** Effect of Water extract of* C. molmol* and DOX on the Levels of AFP, TAC and TNF-ἁ

	Parameters					
Groups	AFP (IU/L)		TAC (nmol/L)		TNF-ἁ (ng/ml)	
	Mean ± SE	% change	Mean ± SE	% change	Mean ± SE	% change
Control	0.53±0.18	…….	0.760±0.015	………	38.5±0.5	……
DEN	4.3±0.26*	711.30%	0.185±0.07*	-75.65%	48.25±1.2*	25.30%
DEN + DOX	3.13±0.14^#^	-27.20%	0.175±0.025^#^	-5.40%	54.15±0.8^#^	12.20%
DEN + Myrrh 250	3.8±0.08^#^	-11.60%	0.35±0.005^#^	89.20%	56.5±1.5^#^	17.10%
DEN + Myrrh 500	3.1±0.23^#^	-27.90%	0.41±0.03^#^	121.60%	44.5±2.5^#^	-7.70%

Data are expressed as Mean ± SE (n = 10 rats), Means with different superscript letters are significantly different at p < 0.05. Data are analyzed with one-way ANOVA followed by the Dunnett test at p < 0.05. *significant difference from control group and ^#^ significant difference from induction with DEN group at p < 0.05

**Table 3:** Effect of Water Extract of *C. molmol *and DOX on the Levels of Oxidative Stress MDA and GSH in Liver Tissue

	Parameters			
Groups	MDA (nmol/g. tissue)		GSH (nmol/g. tissue)	
	Mean ± SE	% change	Mean ± SE	% change
Control	24.5±0.5	…….	13.37±0.24	………
DEN	50.77±0.9*	107.22%	6.5±0.17*	-51.38%
DEN + DOX	30.43±0.5^#^	-40.06%	8.4±0.05^#^	29.23%
DEN + Myrrh 250	23.37±0.2^#^	-54%	11.37±0.3^#^	74.90%
DEN + Myrrh 500	25.87±1.1^#^	-49.04%	13.87±0.27^#^	113%

Data are expressed as Mean ± SE (n = 6 rats), Means with different superscript letters are significantly different at p < 0.05. Data are analyzed with one-way ANOVA followed by the Dunnett test at p < 0.05. *significant difference from control and ^#^ significant difference from induction with DENA group at p < 0.05

## Discussion

HCC is an autosomal disease, being the fourth most common cause of death from cancer worldwide (Villanueva, 2019). It occurs mostly in people with symptoms of liver disease as liver cirrhosis. Nitrosamines and dietary carcinogens are hazardous to human or animal health that associated with the development of HCC. Meanwhile, many studies revealed that DEN-induced hepatic injury is associated with alteration in the serum enzyme markers (Ahmed et al., 2017, Singh et al., 2018). Following existing data, there is a significant elevation in ALT, AST, and ALP enzyme activities of DEN-treated rats which reflects damage to hepatocytes as a result of tumor growth (Tam, 2013). This increase may be due to hepatic necrosis and consequent outflow of these enzymes from neoplastic cells or injured hepatic cells into circulation or may be due to probable effect of the tumor on adjacent tissues leading to loss of its enzyme content and their release into the blood. These conclusions go in agreement with the results of the current histopathological study which showed several inconstant-sized vacuoles in the hepatocytes, multifocal necrotic lesions of HCC-bearing rats (Abdo et al., 2015). Clinical trials are established on bioactive compounds extracted from natural sources which have a therapeutic effect as anti-inflammatory, anticancer activity by decreasing the incidence of tumor (Ahmed et al., 2017). Myrrh (C .molmol ), one of the most natural medicine, reduced alterations of liver enzyme markers according to concentration manner, this is due to the existence of hepatoprotective natural bioactive ingredients in the extract of Myrrh (Alqahtani et al., 2020) which can reduce free radical-induced hepatic injury as well as help in hepatocyte restoration (Dolara et al., 2000), improved the hepatic structure and function (Khalil et al., 2020), inhibit liver injury, continued cell membrane stabilization/integrity and consequently blocked the enzyme release (El-Shahat et al., 2012).

AFP is an important serum indicator of HCC diagnosis (Charrière et al., 2016). It suppresses apoptosis, stimulates cell proliferation, and acts as an immunosuppressive mediator (Mizejewski, 2013). The increased level of AFP detected in DEN-induced animals is a hallmark of HCC as stated before by Jagan et al., (2008). In the same line with our result, Kadasa et al., (2015); Mansour et al., (2019) reported a significant elevation of AFP in DEN-treated rats compared to the control group. Herein, AFP showed a decrease in *C. molmol* treated group compared to DEN rats in a dose-dependent manner. This amelioration may be due to the ability of the Myrrh component to arrest the proliferation of cancer cells as reported in vitro before (Chen et al., 2013; Alqahtani et al., 2020). Another serum marker, TNF-α, is considered a vital inflammatory mediator of proinflammatory cytokines induced by monocytes and macrophages through inflammation (Ding et al., 2019 ) and as inflammatory responses and induction of apoptosis (Panasiuk et al., 2006). In the present study, there was a significant increase in serum TNF-α in DEN-induced HCC animals. According to the previous studies, we found that DEN-induced HCC in rats led to an increase in serum TNF-α levels (Song et al., 2013). This result was in harmony with histopathological alterations as inflammatory accumulation is a hallmark of central hepatocellular necrotic lesions detected in liver damage (Geiger-Maor et al., 2015). While the decrease of TNF-α level in Myrrh group rats in a dose-dependent manner may be due to its anti-inflammatory and anti-cancer activity (Steinman et al., 2017 and Shoemaker et al., 2005) respectively. 

Carcinogenesis occurs when there is an imbalance between oxidative stress and the antioxidant defense system (Tsai et al., 2009). In this study, The DEN-induced rats showed an increase in MDA level that has a cytotoxicity effect and depletion in TAC and GSH levels, this may be due to that the DEN induces liver dysfunction associated with the generation of reactive oxygen species (ROS), membrane lipid peroxidation and therefore alterations in antioxidant defense mechanisms (Elguindy et al., 2018; Singh et al., 2018). Furthermore, the current study showed a significant improvement in oxidative stress through a decrease in the activities of MDA antioxidant enzyme in the liver of animals treated with Myrrh with a dose in a dependent manner with an increase of TAC and GSH in liver tissue as reported by Seifried et al., 2003 who stated that there was a great association between consumption of antioxidant products and reduced the incidence of cancer.

Also, treatment with DEN induced cell proliferation and increase in N/C ratio which is associated with the DNA destruction, mutations, and induction of HCC as stated before by Umemura et al., (2003). On the other hand; the groups treated with two doses of Myrrh showed renovation of normal hepatic architecture with some vacuolar variations. Likewise, administration of rats with chemotherapy drug doxorubicin for the comparison of the effectiveness of Myrrh revealed fewer pathological changes induced by DEN (Rashed et al., 2020; Zeng et al., 2020). 

Apoptosis is a programmed cell death concerning the degradation of cellular constituents by a group of cysteine proteases called caspases (cysteinyl aspartate-specific proteases) (Elmore 2007; Ghavami et al., 2009).In the present study, there is a high expression of Caspase-3 in the DEN group as a hepatocarcinogen agent due to DNA destruction. On the other hand; administration of either dose of Myrrh to DEN treated animals indicated the low expression of caspase3, this result confirms that Myrrh was able to reduce hepatocarcinogenic features in HCC-bearing rats. Alqahtani et al., 2020 stated that Myrrh prevents cell proliferation in the S phase and caused a significant G2/M arrest, then stops the cancerous cell cycle. 

VEGF is the central growth factor and the main regulator facilitating the angiogenesis of HCC (Bogusławska-Duch et al., 2020). Basa et al., (2011) stated that the immunohistochemical expression of VEGF is increased in 87.7% of HCC cells. Data in the present study displayed that the immunohistochemical expression of VEGF in the DEN induced group was significantly increased compared to the control and treated Myrrh groups as stated before by Mahmoud et al., (2017); Arboatti et al., (2018); Alqahtani et al., (2020). This may be due to the high angiogenic activity in DEN-induced hepatocarcinoma in rats by stimulating vascular walls breaking that induce protease synthesis and act on endothelial cells leading to the formation of new blood vessels and capillarization of adjacent normal existing ones, thus promoting tumor growth and tumor stroma formation Ding et al., (2017). On the other hand; the administration of Myrrh showed low expression of VEGF in a dose-dependent manner. Our result is in harmony with Khalil et al., (2020) who stated that Myrrh shows a potential anti-angiogenic and anti-metastatic effect as a result of its role in inhibiting endothelial matrix metalloproteinase-2 (MMP-2) which is responsible for the degradation of the basement membrane of blood vessels and scavenging of free radical. This concept was also verified through the assessment of Myrrh on the TGF- β1 expression, another angiogenic factor. The immunohistochemical analysis revealed that TGF-β1 positive cells significantly increased in DEN groups. The over-expression of TGF-β1 can induce hepatic fibrosis and eventually end-stage liver disease which supports our findings as TGF-β1 is a significant peptide mediator of hepatic stellate cells (HSC), which activate and stimulate matrix synthesis, leading to progressive liver fibrosis (Dewidar et al., 2019). While TGF-β1 positive cells significantly reduced in treated groups which specify that Myrrh suppresses DEN induced hepatocarcinogenesis by its inhibitory effects on HSC activation through suppressing TGF-β1 expression (El-Shahat et al., 2012).

CEA tumor marker is a glycoprotein that acts as a cell adhesion factor and is increased in the circulation due to a weakness of cell connection through tumor growth (Yoshikawa et al., 2017, Ibrahim et al., 2020). Subsequently, CEA typically occurs at very low levels in the blood. Nevertheless, the current immunohistochemical findings revealed that the administration of DEN significantly increases the expression of CEA, because it can be used as a tumor marker as reported before by (Fathy et al., 2017). Remarkably, treatment of the DEN-induced rats with Myrrh in a dose-dependent manner significantly reduced the expression of CEA as reported before by (Mansour et al., 2019).

In conclusion, Myrrh as an anti-carcinogenic and anti-angiogenic agent plays a vital role in reducing DEN-induced hepatocarcinogenesis. It possessed a worthy and promising therapeutic effect due to the improvement in the tumor biomarkers as AFP, TNF-α, Caspase- 3, VEGF, TGF-β1, CEA, and reduction of the lipid peroxidation. This will encourage the conduction of additional studies to determine specific mechanisms of this effect. Furthermore, these findings will open new perceptions for the use of Myrrh only or in combination with other chemotherapeutic agents to inhibit, slow, or reverse the incidence of liver cancer, one of the greatest public malignancies worldwide.

## Author Contribution Statement

H.M.A. conceived the idea of the work, carried out the experiment and biochemical analysis. A.M.M. performed the analysis and planned the experiment. A.A.O. and A.A.S.A. carried out the histological and immunohistochemical examination and designed the figures. All authors discussed the results and contributed to the final manuscript, analyzed the data and wrote the manuscript. All authors have read and agreed to the published manuscript.
